# Scrub typhus among patients with acute febrile illness in Bhutan: A cross‐sectional study

**DOI:** 10.1002/puh2.98

**Published:** 2023-06-08

**Authors:** Karma Norbu, Karma Dema, Kuenga Choden, Tshewang Gyeltshen

**Affiliations:** ^1^ Department of Pathology and Laboratory Medicine Yebilaptsa Hospital Zhemgang Bhutan; ^2^ Department of Dental Surgery Tsirang Hospital Damphu Bhutan; ^3^ Graduate School of Public Health St. Luke's International University Tokyo Japan

**Keywords:** acute febrile illness, Himalayas, mites, *Orientia tsutsugamushi*, rickettsia, scrub typhus, zoonoses

## Abstract

**Introduction:**

Early diagnosis and treatment plays an important role in preventing mortality in scrub typhus infection. In districts situated in the subtropical region of Bhutan, scrub typhus remains an important aetiology among those with acute febrile illness. Zhemgang is one district at 572 m above sea level with warm humid climate, and the majority of population are involved in farming and livestock rearing.

**Methods:**

This was a cross‐sectional study among patients with acute febrile illness in Zhemgang district, Bhutan, tested for scrub typhus rapid diagnostic kits. Variables were extracted from the laboratory registers for tests conducted between January 2019 and December 2020. Risk factors associated with scrub typhus were assessed using logistic regression. This study was approved by the Research Ethics Board of Health, Bhutan.

**Results:**

There were 922 tests conducted for scrub typhus. The test positivity rate was 8.2% (*n* = 76) with the highest reported in Panbang Hospital (43, 56.6%) followed by Yebilaptsa Hospital (27, 35.5%) and Zhemgang Hospital (6, 7.9%). A higher number of cases were detected in females (44, 57.9%) and in the month of September (17, 16.3%). The factors associated with scrub typhus positivity rates were hospitals located in lower altitudes, age, sex and seasons (spring, summer, autumn and winter).

**Conclusions:**

One tenth of patients with acute febrile illness tested positive for scrub typhus with the adequate volume of tests prescribed by the clinicians. Our study shows that summer months recorded higher proportions of scrub typhus infection.

## Graphical abstract

Leptotrombidium pallidum, a vector of scrub typhus

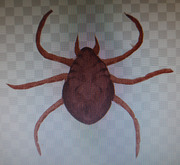

## INTRODUCTION

Scrub typhus affects one billion people globally with one million new cases occurring annually [1]. Scrub typhus ranks among the highest burdens in the developing countries [2]. The disease is transmitted by infected chigger mites. The diagnosis of scrub typhus is based on clinical manifestations, environmental exposure and epidemiological clues. It is characterized by fever, nausea, rashes, vomiting, myalgia, lymphadenopathy, eschar, cough, headache, gastrointestinal symptoms and dyspnoea. Pleural effusion, hepatomegaly, oedema, acute kidney injury, pneumonitis, acute respiratory distress syndrome and meningitis may occur in untreated patients. Fatality rates range from 6% to 35% in untreated and missed diagnoses [2, 3].

Geographically, the highest incidence of scrub typhus is reported in subtropical climate and is endemic in Taiwan, Pakistan, Afghanistan, Maldives, Bangladesh, India and Japan [4]. Outdoor activities, agricultural works and living near grassland and fields are associated with the high risk of exposure [5]. Scrub typhus is the leading infectious diseases in northern India during monsoon seasons, and transplacental transmission of scrub typhus was also reported in a pregnant woman who has delivered a preterm baby with other multi‐organ failure [6]. In Bhutan, the case was first reported in Gedu in 2009. Gedu is situated at around 1520 m above sea level; climatic condition varies from hot and wet subtropical in southern parts to cool and dry weather in the northern part. An outbreak was reported in 2014 at the Singye Namgyal Primary School in Wangdue Phodrang district and claimed two lives [7].

The prevalence of scrub typhus in Bhutan is 62 per 100,000 population at risk with highest incidences in the southern districts with subtropical climate, high agricultural production and in rural residents with farming occupation [8]. According to the study conducted in Bhutan in 2020, patients’ visit and admission in the hospitals of southern districts of Bhutan had increased due to dengue and scrub typhus infections [9]. In order to provide quality patient care and to provide appropriate empirical treatments, more acute febrile illness studies from the subtropical region of Bhutan are needed [8, 9].

In this study, we investigated the seasonal pattern of occurrence of laboratory‐confirmed scrub typhus among patients with acute febrile illness treated in the three hospitals in Zhemgang district, situated in the central part of Bhutan.

## METHODS

### Study design and study setting

This was a cross‐sectional study using laboratory registers in three hospitals in Zhemgang district, Bhutan. Zhemgang is located in the subtropical region at an altitude of 572 m above sea level. Summer months are warm with rain, and winter months are generally pleasant with little to no rainfall. The average annual temperature in the district is 16.1°C. The district had a population of 17,763 in the 2017 census with the majority involved in agriculture farming and livestock rearing. It has 12 primary health care centres, 4 sub‐posts and 3 hospitals in Yebilaptsa, Panbang and Zhemgang. The three hospitals are equipped with laboratory services to diagnose scrub typhus. Zhemgang is located 304 km by road from the country's capital city: Thimphu. Yebilaptsa Hospital is 35 km from the district administration centre and lies 500–620 m above sea level. It is extremely hot in summer and cold in winter. Panbang is situated at 230–1500 m above sea level. It experiences hot and humid weather with an average room temperature of 35°C in summer months and 20°C in winter months.

### Study variables

Variables extracted from the registers included the age and sex of patients, the name of the hospital and the dates when tests were conducted for the period between January 2019 and December 2020. Laboratory results were conducted with SD Bioline Tsutsugamushi Test rapid diagnostic test kits in 2019 and SD Bioline Tsutsugamushi in 2020. The SD Bioline Tsutsugamushi Test rapid test kit has a sensitivity of 99% and a specificity of 96%; the Standard Q Tsutsugamushi IgM/IgG rapid test kit has a sensitivity of 96.6% and 98.6% specificity [16].

### Data analysis

The data were entered into Microsoft Excel 2016 and exported as a CSV file for analysis. Data analysis was carried out in R using the mStats package. Categorical variables are summarized as frequencies and percentages, and continuous variables are summarized as means and standard deviations. Information on the key characteristics of tests prescribed is summarized based on the age, sex, months/seasons and hospitals. We identified predictors of positive results using a stepwise backward elimination multi‐variable logistic regression model, fitted with all the predictor variables and the final model selected based on the lowest Akaike information criterion score.

## RESULTS

**FIGURE 1:**
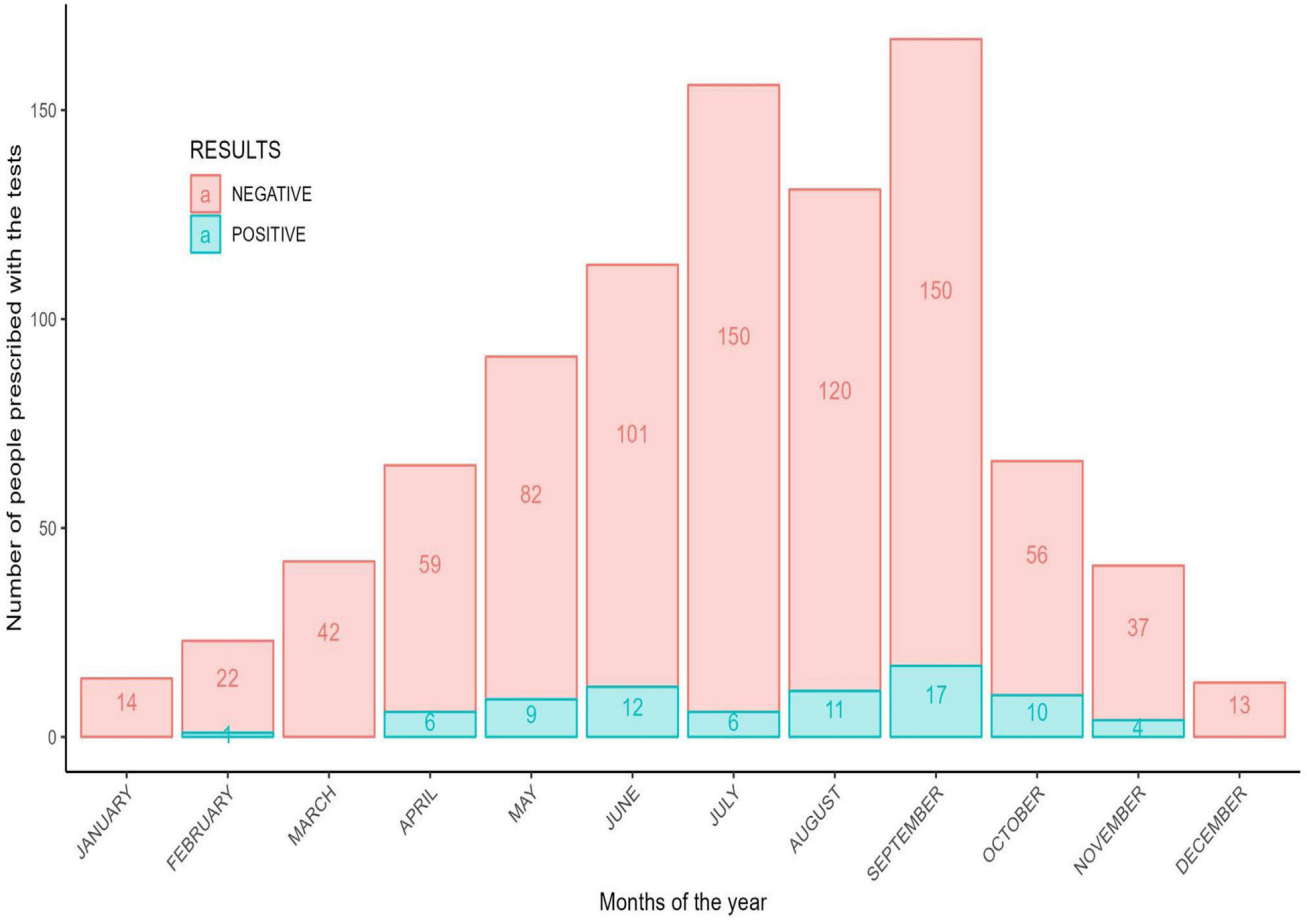
Pattern of test positivity for scrub typhus across the months of the year in patients with acute febrile illness in Zhemgang district, Bhutan, 2019–2020.

A total of 922 tests to scrub typhus were conducted in the 3 hospitals. The mean age of the patients was 36.1 (±SD 17.9) years, range 1 month to 94 years. The majority of the tests were prescribed in summer months (June–September). The proportion of laboratory‐confirmed test positivity was 8.2% (*n* = 76). There were 470 (51.0%) females. The test positivity rates were 56.6% (*n* = 43) in Panbang Hospital, 35.5% (*n* = 27) in Yebilaptsa Hospital and 7.9% (*n* = 6) in Zhemgang Hospital. The test positivity rate was higher in the month of September. The basic description of the test prescriptions is shown in Table 1. The scrub typhus test positivity rates across months are shown in Figure 1.

**TABLE 1:** Description of scrub typhus rapid kit tests performed in patients with acute febrile illness in Zhemgang district, Bhutan, 2019–2020.

Variables	Total	Scrub typhus positive	
		*n*	%
Sex			
Female	470	44	9.4
Male	452	32	7.1
Age group (years)			
0–10	99	5	5.1
11–21	131	10	7.6
22–32	185	8	4.3
33–55	336	29	8.6
≥56	171	24	14.0
Hospitals			
Panbang Hospital	324	43	13.3
Yebilaptsa Hospital	446	27	6.0
Zhemgang Hospital	152	6	4.0
Year			
2019	574	68	11.9
2020	348	8	2.3
Seasons			
Autumn (Sept–Nov)	120	27	22.5
Spring (Mar–May)	269	15	5.6
Summer (Jun–Aug)	454	30	6.6
Winter (Dec–Feb)	79	02	2.5

The odds of testing positive of scrub typhus are three times higher in Panbang Hospital compared to Yebilaptsa Hospital with *p* < 0.001 (95% CI, 1.79–5.19). Age is a strong predictor for the positive scrub typhus test. For each 1‐year increase in the age of the patient, the odds of testing positive for the scrub typhus increased by 1 with *p* < 0.001 (95% CI, 1.0). The change of season is not associated with positive test results. The details of the regression analysis are provided in Table 2.

**TABLE 2:** Factors associated with laboratory‐confirmed scrub typhus among patients with acute febrile illness in Zhemgang district, Bhutan, 2019–2020.

	Odds ratio	95% CI	*p* value
Hospitals (Ref Zhemgang Hospital)			
Yebilaptsa Hospital	1.35	0.56–3.82	0.531
Panbang Hospital	3.05	1.79–5.19	<0.001
Age	1.02	1.01–1.04	<0.001
Sex (Ref female)			
Male	0.72	0.44–1.17	0.185
Seasons (Ref winter)			
Spring (Mar–May)	0.79	0.39–1.62	0.526
Summer (Jun–Aug)	0.64	0.31–1.29	0.208
Autumn (Sept–Nov)	12.80	2.44–236.00	0.016

## DISCUSSION

The proportion of laboratory‐confirmed scrub typhus was low compared to 12% reported in 2016 and 470 positive cases reported in 2019 in Bhutan, and 67.1% reported in Chitwan, Nepal in 2016 [7, 8]. Many regions of Bhutan have environmental, climatic and occupational risk factors for the transmission of scrub typhus [16]. Zhemgang is the remotest and the least developed district in the country with 41.4% of its population under poverty. Panbang Hospital is located at lower altitude compared to Yebilaptsa and Zhemgang Hospitals. It is a hot and humid place and share border with the Indian state of Assam.

Scrub typhus infection is a public health problem in Bhutan. It remained silent after the first case in 2008. Data‐based research from 2009 to 2014 found that a number of positive results increased over the years with the trend of seasons [7]. More female tested positive in our study concurs with a study conducted in Bhutan, May 2023 [16], the Himalayan region of India [11] and that of Sri Lanka [12]. In rural Bhutan, females usually engaged more in farm and livestock works than males, which could be a possible explanation for this difference.

The seasonal variation in positive cases although statistically not a significant predictor but was shown to be higher in autumn and summer months which are similar to that reported in Assam, India [13], southern China [14] and Bhutan [16]. Summer and autumn are the seasons for major farm works in rural Bhutan, which lead to increased exposure to outdoor activities. These are also the months of high rainfall with humid and hot climate compared to other seasons [10, 16].

Through the years 2010–2014, knowledge about scrub typhus was reported to be low among the health care workers in Bhutan [7]. Our study shows high proportions of tests prescribed compared to the positive case, which is a good indicator of screening the diseases for the early detection. Other acute febrile illnesses such as dengue and other rickettsial diseases cause clinical features similar to scrub typhus infection [15]. Physicians need to be aware of the disease and screening all the patients for early diagnosis and better disease outcomes. Availability of the rapid test kits is only at hospital level but supplying till basic health unit level will enhance the detection of the disease as early as possible.

The programmatic approach to disease awareness, disease pathology and disease diagnosis must be reinforced among prescribing physicians. The field implementations must be enhanced in geographically and occupationally high risks of exposure groups.

One limitation of our study is that the laboratories used two different commercial rapid test kits within the 2 years, and both test kits could not be compared to indirect immunofluorescence assay and enzyme‐linked immunoassay testing.

## CONCLUSION

One tenth of patients with acute febrile illness tested positive for scrub typhus. This proportion is low compared to findings from the region. Half of cases that tested positive were detected in the months of June–September. The volume of tests prescribed demonstrates awareness among clinicians about the early detection of the disease.

## AUTHOR CONTRIBUTIONS

Karma Norbu conception/design acquisition of data, manuscript drafting, giving approval for the final version to be published. Tshewang Gyeltshen conception/design of the protocol, data analysis/interpretation, manuscript drafting/critical review of the paper and the approval of the final version to be published. Kuenga Choden and Karma Dema critically reviewing the paper and giving approval for the final version to be published. All authors have reviewed the manuscript.

## CONFLICT OF INTEREST STATEMENT

The authors declare that there is no conflict of interest that could be perceived as prejudicing the impartiality of the research reported.

## ETHICS STATEMENT

The ethics statement was obtained from the Research and Ethics Board of the Ministry of Health, Royal Government of Bhutan, Ref. No. *REBH/Approval/2021/079dated 05/07/21*. A waiver of consent has been sought from the committee and has been granted based on the study design. All methods were carried out in accordance with relevant guidelines and regulations as enshrined in Helsinki Declarations 1964.

## Data Availability

Data and materials available from the authors upon request.
